# Pay-it-forward intervention increased pneumococcal vaccine uptake among older adults in China: a randomized controlled trial

**DOI:** 10.1186/s12916-026-04624-2

**Published:** 2026-01-19

**Authors:** Jiao Qin, Chunxing Tao, Ting Huang, Liangjia Wei, Jinfeng He, Ruby Congjiang Wang, Dan Wu, Shiyu Qin, Qiuqian Su, Yanxiao Gao, Shuiming Chen, Ganqin Wang, Zhifeng Lin, Xinju Huang, Xianyan Tang, Chuanyi Ning, Hao Liang, Weiming Tang, Salma Gayed, Jason J Ong, Junjun Jiang, Li Ye, Joseph D Tucker, Bingyu Liang

**Affiliations:** 1 Guangxi Key Laboratory of AIDS Prevention and Treatment, School of Public Health & Life Sciences Institute, Guangxi Medical University, Nanning, Guangxi 530021 China; 2 Youjiang Medical University for Nationalities, Baise, Guangxi 533000 China; 3 SESH Global, London School of Hygiene and Tropical Medicine, London, UK; 4 Department of Social Medicine and Health Education, School of Public Health, Nanjing Medical University, Nanjing, Jiangsu 211166 China; 5 Shenzhen Institute of Advanced Technology, Chinese Academy of Sciences, Shenzhen, Guangdong China; 6 Jinzhou Community Health Service Center, Zintou Street, Qingxiu District, Nanning, Guangxi 530021 China; 7 Panlong West Community Health Service Center, The Second People’s Hospital of Nanning, Liangqing District, Nanning, Guangxi 530206 China; 8 University of North Carolina Project-China, Guangzhou, China; 9 Cambridge University NHS foundation Trust, Cambridge, United Kingdom; 10 Melbourne Sexual Health Centre, Alfred Health; The School of Translational Medicine, Melbourne, VIC Australia; 11 Clinical Research Department, London, School of Hygiene and Tropical Medicine , London, UK; 12 Department of Medicine, University of North Carolina at Chapel Hill, Chapel Hill, NC USA

**Keywords:** China, Older adults, Pay it forward, Pneumococcal vaccine, Cost

## Abstract

**Background:**

Pneumococcal vaccination reduces morbidity and mortality among older adults, yet coverage remains suboptimal in China. This study aimed to assess the effectiveness of a pay-it-forward intervention (covering two-thirds of the pneumococcal vaccination cost and offering the option to donate) in increasing pneumococcal vaccination among older adults (aged 60 years or older) in China, compared to standard-of-care self-paid vaccination.

**Methods:**

We used block randomization (block size = 4) to assign participants to a pay-it-forward arm and a standard-of-care arm in a 1:1 ratio. The primary outcome was pneumococcal vaccination. Secondary outcomes included influenza vaccine uptake, vaccine confidence, successful vaccine referral, and cost-effectiveness. Logistic regression analysis was used to compare PPSV-23 and influenza vaccination coverage and vaccine confidence between the two groups. The cost-effectiveness of the interventions was assessed using a micro-costing approach from the healthcare provider’s perspective.

**Results:**

From January to September 2024, 221 older adults were randomized (110 in the pay-it-forward group and 111 in the standard-of-care group). Pneumococcal and influenza vaccine uptake were significantly higher in the pay-it-forward arm (70.9% and 30.0%) than in the standard-of-care arm (13.5% and 17.1%), with adjusted odds ratios of 17.20 (95% *CI*, 8.39–37.60) and 2.29 (95% *CI*, 1.17–4.65). The pay-it-forward group also exhibited greater confidence in the safety (4.29, 95% *CI*, 1.78–11.50), importance (5.15, 95% *CI*, 2.05–14.60), and effectiveness (7.14, 95% *CI,* 2.36–27.50) of the vaccine than that in the standard-of-care group. The pay-it-forward group had a higher successful vaccine referral rate (15.5% vs. 10.8%) and had a lower economic cost per person vaccinated (US $95.67 vs. US $278.56) compared with the standard-of-care arm.

**Conclusions:**

Our findings demonstrate that the pay-it-forward intervention significantly enhances pneumococcal and influenza vaccination coverage and improves vaccine confidence among the older adults. This study highlights the potential of the pay-it-forward intervention as an effective means to boost health service utilization.

**Trial registration:**

2024–01-03, Chinese Clinical Trial Registry, ChiCTR2400079410.

**Supplementary Information:**

The online version contains supplementary material available at 10.1186/s12916-026-04624-2.

## Background

Pneumonia is a severe global respiratory infection associated with significant morbidity and mortality [1], especially among vulnerable groups like young children and older adults. Due to age-related declines in mucociliary clearance and immune function [2, 3], older adults are at increased risk of community-acquired pneumonia (CAP) and pneumococcal diseases (PDs), resulting in higher hospitalization and mortality rates that impose a substantial healthcare burden [4]. According to estimates from the Global Burden of Disease Study, pneumococcal lower respiratory infections caused nearly half a million deaths among adults aged ≥ 70 years, accounting for 45.74% of global pneumococcal infection deaths [5]. In China, the estimated incidence of CAP in 2018 ranged from 29.8 to 221.0 per 10,000 individuals, with 37% of cases occurring in those aged ≥ 65 years [6].

To prevent pneumonia, vaccination is widely implemented in China. The 23-valent pneumococcal polysaccharide vaccine (PPSV-23) is the primary option for preventing *Streptococcus pneumoniae*, the primary causative agent of the illness [7]. PPSV-23 covers 23 prevalent serotypes that account for 65%–91% of invasive pneumococcal disease cases in adults worldwide, providing broad protection [8]. The World Health Organization (WHO) and the Chinese Center for Disease Control and Prevention recommend the pneumococcal vaccine for adults aged 65 and above [9]. The PPSV-23 vaccine is 70% effective against invasive PDs [10] and 30.9% effective against CAP [11], leading to significantly fewer hospitalizations among older adults. A single dose provides approximately 5 years of protection, significantly reducing the risk of pneumonia and healthcare costs [9]. Despite these clinical benefits, the vaccine remains an out-of-pocket expense in China, resulting in low vaccination coverage among older adults, ranging from 1 to 42% [12].

Government efforts to increase the uptake of PPSV-23 include subsidy programs [13], awareness campaigns, and recommendations from healthcare providers [14], but these measures have not significantly improved coverage among older adults in China. Even with subsidy programs or free vaccinations, the uptake ranges from 30% to 42.1% [13, 15], well below the over 60% coverage seen in developed countries [16, 17]. Barriers include financial constraints [18], low vaccine confidence [19], lack of awareness [20], insufficient health education [12], and inadequate community engagement [21]. These challenges highlight the importance of developing and implementing innovative strategies to increase pneumococcal vaccinations among older adults.

Pay it forward is an innovative social intervention rooted in behavioral economics and the theory of upstream reciprocity [22]. Behavioral economics posits that human decision-making often deviates from perfect rationality [23], while the upstream reciprocity theory suggests that recipients of a benefit are inclined to “pay it forward” to others rather than directly repaying the benefactor [24]. This concept leverages social norms, fairness preferences, and prosocial motivations to lower barriers and encourage participation in collective actions [25, 26]. Beyond public health, pay it forward has been applied in education [27], charitable giving [28], and bill payment [29], demonstrating how small acts of generosity can initiate cascades of prosocial behavior. In China, pay it forward has been successfully implemented to promote health behaviors such as vaccine uptake [30] and sexually transmitted infection testing [22, 31]. However, the majority of studies on this intervention have focused on urban areas [32], and only a limited number have thoroughly assessed its effectiveness in the context of pneumococcal vaccination among older adults in resource-limited settings. This study employed a two-arm, parallel, randomized controlled trial to evaluate the effectiveness of the pay-it-forward intervention in increasing pneumococcal vaccination uptake among people aged 60 years and above in Nanning City, Guangxi, China.

## Methods

### Study design and setting

This two-arm, parallel, randomized controlled trial was conducted to evaluate the effectiveness of the pay-it-forward intervention in promoting the uptake of PPSV-23 among older adults. The study was conducted in four community health service centers (CHSCs) in Nanning, Guangxi, China: Jinzhou, Tanluo, Panlong West, and Poyang. These CHSCs provide essential primary care services for community residents. Recruitment began on January 3, 2024, after confirmation of vaccine availability by the local study sites, and concluded on September 30, 2024. This study protocol was previously published in *JMIR Research Protocols* [33].

### Community engagement

Before participant recruitment, the research team conducted project design workshops with community health workers (CHWs) to finalize the study protocol, the recruitment and data collection procedures, the follow-up processes, and the vaccination services. We also invited experienced researchers who had implemented pay-it-forward interventions (e.g., for influenza and HPV vaccination and STI testing) to share insights. CHWs supported all stages of the study—assisting in recruitment, co-developing educational leaflets, refining the survey, and suggesting engagement strategies. Participants were encouraged to donate, write handwritten postcards to unvaccinated individuals, and recommend PPSV-23 vaccination to others.

### Study participants and sample size

The inclusion criteria for participants were as follows: (1) Residence in the community for the past 3 months, (2) age of 60 years or older, (3) meeting the medical criteria for PPSV-23 vaccination, and (4) capacity to provide informed consent for vaccination. The exclusion criteria included the following: (1) Suffering from a severe chronic or mental illness and (2) having received a PPSV-23 vaccination in the past 5 years. Only one older adult per household was allowed to participate in this study to prevent clustering. All eligible CHSC participants were invited to participate in the survey by the CHWs or research team members. The sample size was estimated at 204 participants, with detailed procedures outlined in the study protocol [33].

### Randomization and masking

This study utilized block randomization with a block size of 4. Each set of four random numbers was grouped into a block and numbered sequentially as they were generated. Within each block, the two smallest numbers were assigned to the standard-of-care arm, while the two largest numbers were assigned to the pay-it-forward arm. Opaque envelopes containing group allocation and promotional materials were distributed to participants in the order of their enrollment. To minimize bias, the following measures were taken:

1. Random number generation and allocation were kept confidential and known only to the study designers.
2. Neither participants nor on-site staff were aware of group assignments before envelope opening.
3. The physician who prescribed PPSV-23 was blinded to group assignment.
4. Data analysts were blinded during statistical analysis.

### Data collection

Participants were required to complete a baseline questionnaire (Additional file 1) covering social-demographic and clinical characteristics, knowledge of PPSV-23, attitudes toward PPSV-23 vaccination, willingness to receive PPSV-23, and motivations or hesitations regarding vaccination, as well as perceptions and acceptance of the pay-it-forward program (only for the pay-it-forward group). The same follow-up questionnaire (Additional file 2) was used for the second-week and fourth-week follow-up surveys: (1) The PPSV-23 vaccination, incidence of adverse events of vaccination, and the reasons for declining the PPSV-23 vaccination, (2) knowledge and attitude toward PPSV-23, (3) influenza vaccination, and (4) recommendation of pneumococcal vaccination to others.

### Procedure

**Fig. 1:**
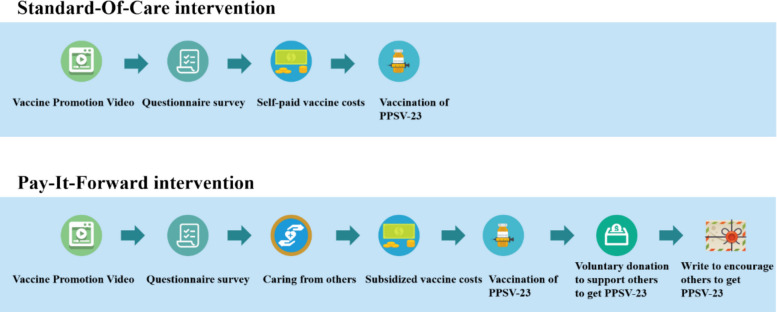
Interventions in the pay-it-forward and standard-of-care arms

Figure 1 compares the interventions in the two groups. In the pay-it-forward arm, participants received leaflets about the PPSV-23 (Additional file 3) and the pay-it-forward program (Additional file 4). They were informed that they could receive a 150 RMB (US $21.28) subsidy for the PPSV-23 vaccination from donations made by the preceding pay-it-forward recipients, with the standard cost being 214–280 RMB (US $30.35–US $39.72). Each participant then watched an introductory video on PPSV-23 and completed a baseline questionnaire with the help of CHWs. Finally, participants were asked about their willingness to be vaccinated and to donate or write a postcard to encourage the subsequent recipients to participate in the pay-it-forward initiative. Donation decisions did not affect subsidy eligibility. Eligible individuals willing to be vaccinated were scheduled for vaccination. Follow-up surveys were conducted at weeks 2 and 4. Donations were collected and managed through a dedicated research account. Each donor was required to note their unique envelope number, and the fund usage was regularly disclosed via WeChat for transparency.

In the standard-of-care arm, participants received only the PPSV-23 leaflet and were informed of the standard vaccination cost (214–280 RMB; US $30.35–39.72). They also watched the PPSV-23 video and completed the baseline questionnaire with CHW support before deciding on self-paid vaccination. Eligible participants could vaccinate immediately or schedule an appointment. Participants in this group were unaware of any information regarding the pay-it-forward program. Follow-up surveys were also conducted at weeks 2 and 4.

### Study outcomes

The primary outcome was defined as the uptake of PPSV-23. This metric was calculated as the proportion of vaccinated individuals within each group relative to the total number of individuals in that group. Secondary outcomes included the following: (1) The uptake of influenza vaccination (participants pay for their vaccination); (2) the cost of each intervention [34]; (3) vaccine confidence, as measured by participants’ perceptions of vaccine importance, vaccine safety, vaccine efficacy, and vaccine management [35, 36]; and (4) successful vaccine referral, defined as participants successfully recommending pneumococcal vaccines to others after participating in the study. Vaccination records were verified through the administrative data.

### Qualitative research

We conducted qualitative research with 13 participants (7 of whom completed the donation and 6 who did not) to better understand their views and acceptance of the donation process, as well as to identify preliminary factors that may influence their donation decision. After obtaining informed consent, a trained researcher conducted face-to-face interviews with each participant, each lasting approximately 20–30 min. All interviews were audio-recorded for subsequent analysis.

### Data analysis

Chi-squared test was employed to describe the participants’ basic characteristics and vaccine recommendation rates. The primary analysis followed the intention-to-treat (ITT) principle, with a sensitivity analysis in the per-protocol (PP) population to assess robustness (Additional file 5). We performed multivariable logistic regression analyses to determine the association between the intervention and the outcomes, including PPSV-23 vaccination and influenza vaccination coverage, as well as vaccine confidence, adjusting all models for basic demographic characteristics. Stratified analyses were conducted by monthly income level (≥ 5000 RMB vs. < 5000 RMB) [37], health status, living status, and area of residence. Firth’s penalized-likelihood logistic regression was applied within each stratum to estimate and compare the adjusted odds ratios for PPSV-23 vaccination coverage between the intervention groups. The results were reported as crude odds ratios (cORs) and adjusted odds ratios (aORs), with 95% confidence intervals (95% CI). All statistical tests were two-sided, with a significance level of *p* < 0.05. R version 4.4.2 was used for the statistical analyses.

We employed a micro-costing approach to evaluate the costs of the standard-of-care and pay-it-forward interventions from the healthcare providers’ perspective. The time horizon was 1 year. We leveraged cost data from invoices, staff self-reports, and government-released salary data [38] and reported all costs in US$ (2023). Implementation costs for each intervention were categorized into start-up costs, fixed costs, and recurrent costs. Start-up costs included training expenses during the initial phase of the project. Fixed costs covered vaccinator salaries, while recurrent costs primarily encompassed staff time for participant recruitment and follow-up, as well as material procurement expenses. The unit costs and utilization frequency of all resources incorporated in the model are detailed in Additional File 6 (Table S5). A decision tree model was developed using TreeAge Pro 2022 to conduct cost-effectiveness analysis, with model robustness verified through both one-way and probabilistic sensitivity analyses. We reported total economic costs (sum of start-up, fixed, and recurrent costs), total financial costs (economic costs minus donations), incremental cost-effectiveness ratios, and per capita vaccination cost for each arm. The cost per person vaccinated was calculated by dividing the total economic cost by the number of vaccinated individuals [30].

All interview recordings were transcribed verbatim and verified. Transcripts were coded systematically in MAXQDA software (version 24). A thematic analysis approach was employed to identify and categorize salient themes and patterns inductively. Coding was conducted by a single researcher, who engaged in regular peer debriefing sessions with the study team to enhance reflexivity and reduce potential bias. Data analysis continued iteratively until thematic saturation was reached, defined as the point at which no new themes emerged from the data.

## Results

### Trial profile

**Fig. 2:**
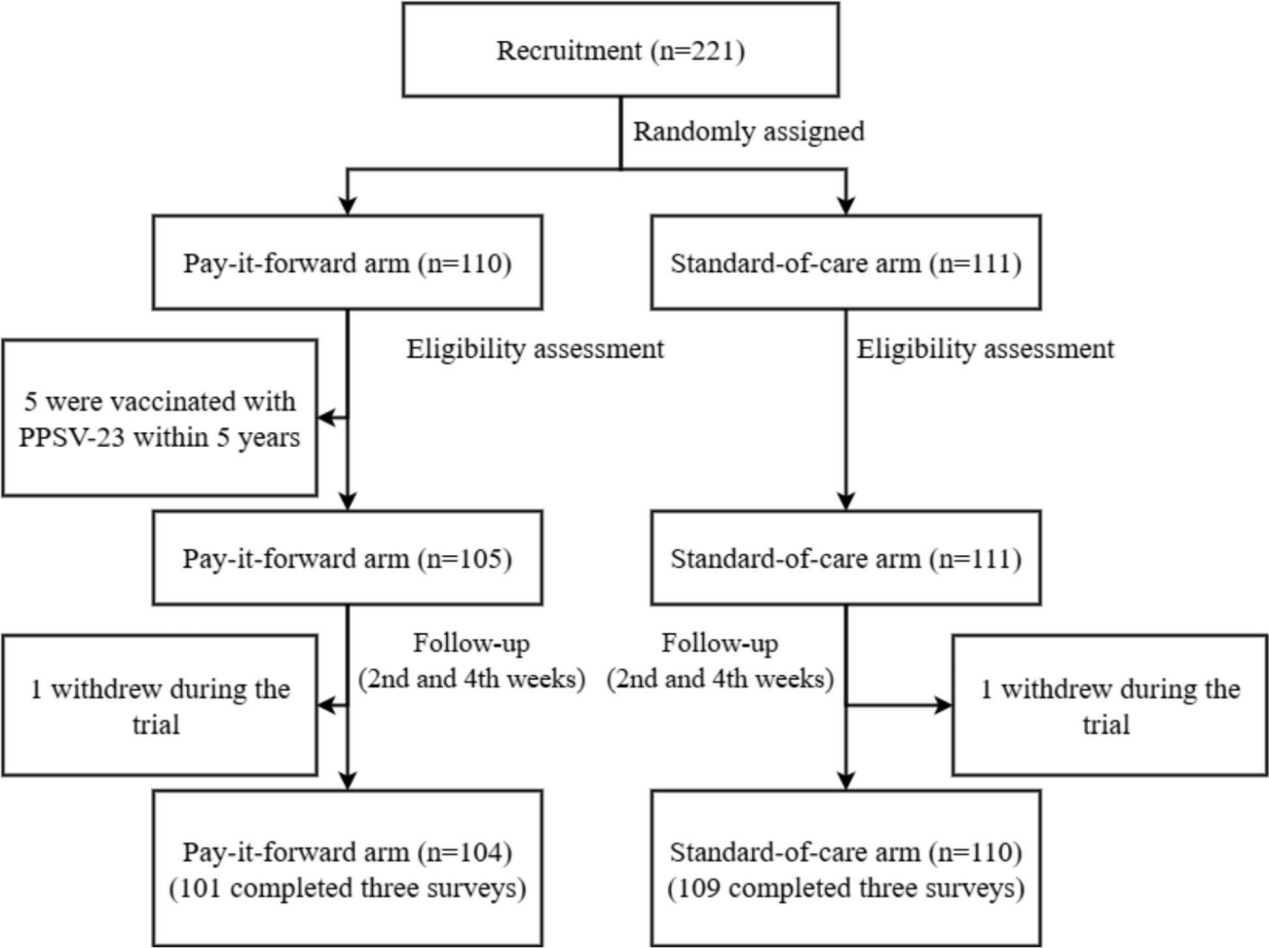
Flow chart of the study

A total of 221 older adults were recruited and randomized to the pay-it-forward arm (*n* = 110) or the standard-of-care arm (*n* = 111) (Fig. 2). After excluding 6 participants from the pay-it-forward arm (5 received the PPSV-23 within 5 years, and 1 dropped out during the trial) and 1 dropout from the standard-of-care arm, 221 participants were included in the ITT analysis and 214 in the PP analysis. To evaluate whether the final analyzed sample (*n* = 221) retained sufficient statistical power, we conducted a post hoc power analysis using the estimated initial effect size (pay it forward: 30%, standard-of-care arm: 10%). With 221 participants and an alpha of 0.05, the calculated power was 96.9%, confirming that the final sample size was sufficient to detect the expected effect.

### Sociodemographic characteristics of the participants

Most sociodemographic characteristics were similar between the pay-it-forward and standard-of-care groups, except for monthly income and living status variables (Table 1). The majority of participants were aged 71 years or older (41.2%), were women (57.9%), were married (81.9%), were retired (73.8%), and were living with a spouse (70.1%). Additionally, 52.50% were rural residents. Monthly incomes varied, with one-third (29.0%) of participants having a monthly income of less than US $276 and 23.1% having incomes exceeding US $690. Furthermore, the majority (69.7%) of participants were living with chronic diseases.

**Table 1:** Sociodemographic characteristics of recruited participants aged ≥ 60 years in Nanning city, China (*n* = 221)

Variables	All	Standard of care	Pay it forward	*p-value*
	***n* = 221**	***n* = 111**	***n* = 110**	
**Sex**				
Male	93 (42.1)	46 (41.4)	47 (42.7)	0.954
Female	128 (57.9)	65 (58.6)	63 (57.3)	
**Age (year)**				
60–65	65 (29.4)	34 (30.6)	31 (28.2)	0.383
66–70	65 (29.4)	28 (25.2)	37 (33.6)	
≥ 71	91 (41.2)	49 (44.1)	42 (38.2)	
**Marital status**				
Married	181 (81.9)	86 (77.5)	95 (86.4)	0.123
Unmarried/divorced/widowed	40 (18.1)	25 (22.5)	15 (13.6)	
**Occupation**				
Farmer	33 (14.9)	22 (19.8)	11 (10)	0.123
Retired	163 (73.8)	77 (69.4)	86 (78.2)	
Others	25 (11.3)	12 (10.8)	13 (11.8)	
**Monthly income (US$)**				
[0.276)	64 (29)	41 (36.9)	23 (20.9)	0.012
[276,414)	58 (26.2)	30 (27)	28 (25.5)	
[414,690)	48 (21.7)	23 (20.7)	25 (22.7)	
≥ 690	51 (23.1)	17 (15.3)	34 (30.9)	
**Living status**				
Living with spouse	155 (70.1)	69 (62.2)	86 (78.2)	0.014
Living without spouses	66 (29.9)	42 (37.8)	24 (21.8)	
**Area of residence**				
Urban	105 (47.5)	48 (43.2)	57 (51.8)	0.254
Rural	116 (52.5)	63 (56.8)	53 (48.2)	
**Distance of residence from the vaccination site**				
Within 1 km	80 (36.2)	35 (31.5)	45 (40.9)	0.295
1–3 km	70 (31.7)	36 (32.4)	34 (30.9)	
≥ 3 km	71 (32.1)	40 (36)	31 (28.2)	
**Smoking in the last 6 months**				
No	189 (85.5)	97 (87.4)	92 (83.6)	0.548
Yes	32 (14.5)	14 (12.6)	18 (16.4)	
**Drinking in the last 6 months**				
No	161 (72.9)	82 (73.9)	79 (71.8)	0.847
Yes	60 (27.1)	29 (26.1)	31 (28.2)	
**Chronic disease**				
No	67 (30.3)	38 (34.2)	29 (26.4)	0.260
Yes	154 (69.7)	73 (65.8)	81 (73.6)	
**Educational level**				
High school and above	100 (45.2)	55 (49.5)	45 (40.9)	0.248
Middle school and below	121 (54.8)	56 (50.5)	65 (59.1)	

### Uptake of PPSV-23 and influenza vaccine

Tables 2 and 3 show that uptake of PPSV-23, and influenza vaccinations were significantly higher in the pay-it-forward group than in the standard-of-care group. A total of 70.9% (78/110) of older adults in the pay-it-forward group received the PPSV-23 vaccine, compared to 13.5% (15/111) in the standard-of-care group. Similarly, 30.0% (33/110) of participants in the pay-it-forward group received the influenza vaccine compared to 17.1% (19/111) in the standard-of-care group. Multivariable logistic regression results showed that compared to the standard-of-care group, participants in the pay-it-forward group had higher odds of receiving the PPSV-23 vaccine (*aOR* = 17.20; 95% *CI*, 8.39–37.60) and the influenza vaccine (*aOR* = 2.29; 95% *CI* 1.17–4.65).

**Table 2:** Multivariate logistic regression of PPSV-23 vaccination uptake in two groups (*n* = 221)

Group	Pneumococcal vaccine		
	**Vaccination, *n* (%)**	**cOR (95% CI)**	**aOR (95% CI)**
**Standard of care**	15 (13.5)	1	1
**Pay it forward**	78 (70.9)	15.60 (8.08, 31.80)	17.20 (8.39, 37.60)
***P* value**		*p* < 0.001	*p* < 0.001

**Table 3:** Multivariate logistic regression of influenza vaccination uptake in two groups (*n* = 221)

Group	Influenza vaccine		
	**Vaccination *n* (%)**	**cOR (95% CI)**	**aOR (95% CI)**
**Standard-of-care**	19 (17.1)	1	1
**Pay-it-forward**	33 (30.0)	2.08 (1.10, 3.99)	2.29 (1.17, 4.65)
***P* value**		*p* = 0.025	*p* = 0.018

For adjusted OR, the model adjusted for age, sex, area of residence, marital status, educational level, occupation, monthly income, living situation, and distance of residence from vaccination site.

For adjusted OR, the model adjusted for age, sex, area of residence, marital status, educational level, occupation, monthly income, living situation, and distance of residence from vaccination site.

**Fig. 3:**
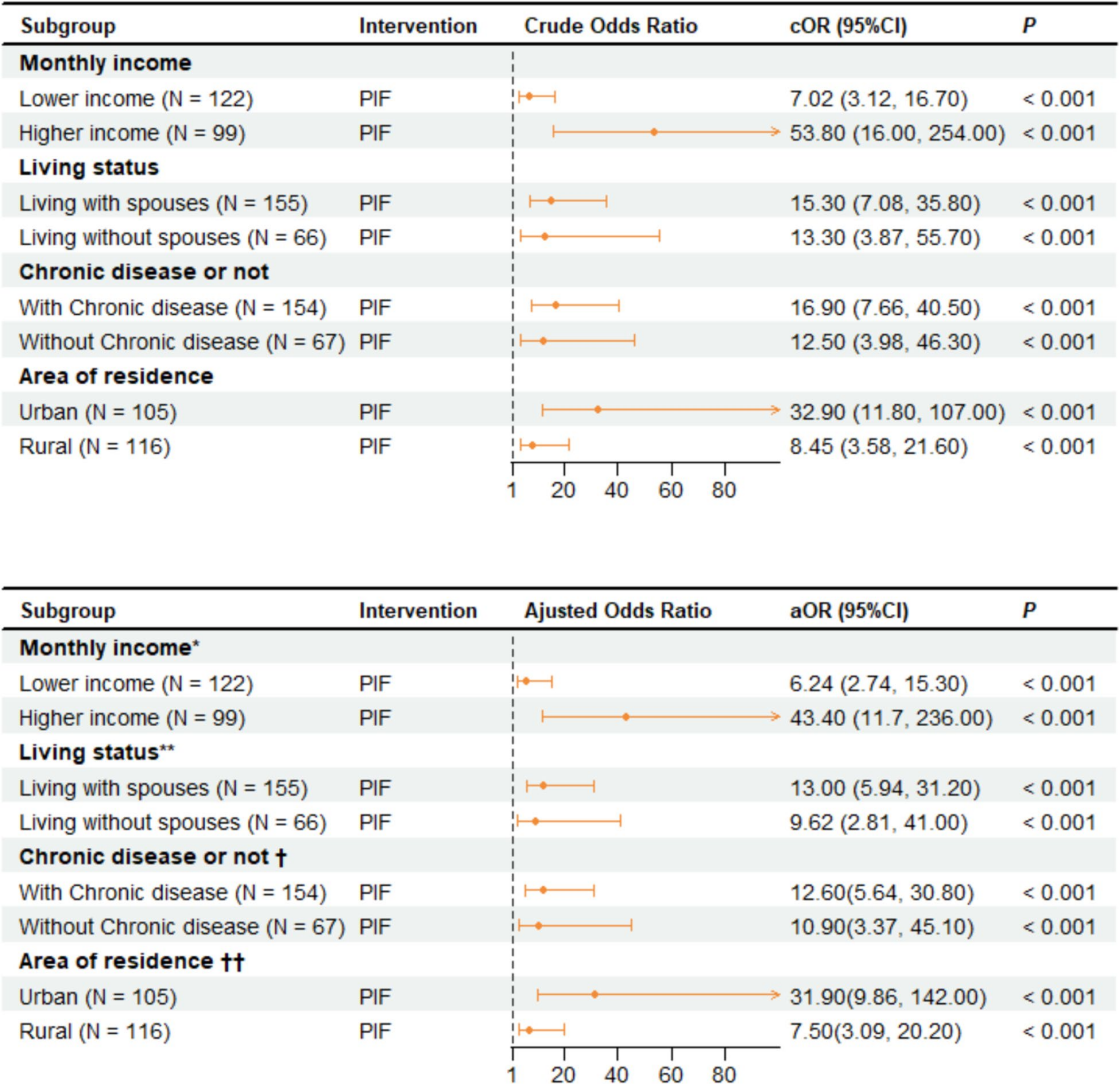
Multivariate logistic regression of PPSV-23 vaccination uptake in the two groups (stratified analysis, *n*** = **221). Note: PIF, pay it forward. Reference: Standard of care. *For adjusted OR, the model adjusted for age, sex, area of residence, marital status, educational level, occupation, living situation, and distance of residence from the vaccination site. **For adjusted OR, the model adjusted for age, sex, area of residence, educational level, occupation, monthly income, and distance of residence from the vaccination site. †For adjusted OR, the model adjusted for age, sex, marital status, area of residence, educational level, occupation, monthly income, living situation, and distance of residence from the vaccination site. ††For adjusted OR, the model adjusted for age, sex, marital status, educational level, occupation, monthly income, living situation, and distance of residence from the vaccination site

Subgroup analyses showed that the effect of the pay-it-forward intervention in promoting PPSV-23 uptake remained significant compared with the standard-of-care group (Fig. 3). The pay-it-forward intervention was found to be more effective in promoting vaccination among those with higher monthly incomes (*aOR* = 43.40; 95% *CI*, 11.70–236.00) compared with those with lower monthly incomes (*aOR* = 6.24; 95% *CI*, 2.74–15.30). The effectiveness of pay it forward in increasing PPSV-23 vaccination was more pronounced among those living with a spouse (*aOR* = 13.00; 95% *CI*, 5.94–31.20) than among those not living with a spouse (*aOR* = 9.62; 95% *CI*, 2.81–41.00). The effectiveness of the pay-it-forward intervention was more pronounced among participants with chronic disease (*aOR* = 12.60; 95% *CI*, 5.64–30.80) than among those without chronic disease (*aOR* = 10.90; 95% *CI*, 3.37–45.10). Compared with rural residents (*aOR* = 7.50; 95% *CI*, 3.09–20.20), pay it forward was more effective in increasing PPSV-23 vaccination among urban residents (aOR = 31.90; 95% *CI*, 9.86–142.00).

#### Secondary outcomes

The rate of successful vaccine referral was higher in the pay-it-forward group than in the standard-of-care group (Table 4), although the difference was not statistically significant (58.6% vs. 41.4%).

**Table 4:** Successful pneumococcal vaccination referrals to others (*n* = 221)

Group	Total	PPSV-23 referral success		*p-value*
		**No**	**Yes**	
**Standard of care**	111 (50.2)	99 (89.2)	12 (10.8)	0.410
**Pay it forward**	110 (49.8)	93 (84.5)	17 (15.5)	

**Fig. 4:**
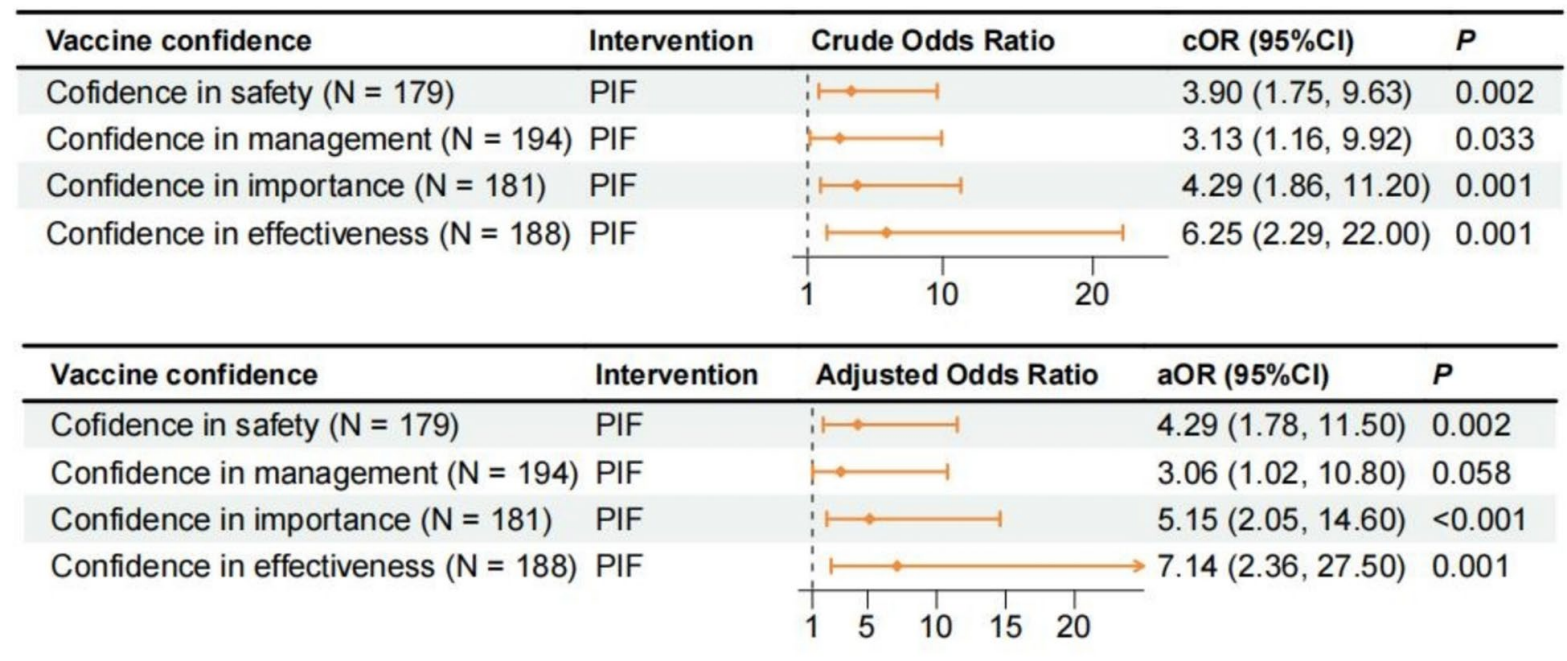
Multivariable logistic regression to compare vaccine confidence between standard-of-care and pay-it-forward groups (*n*** = **214). Note: PIF, pay it forward. Reference: Standard of care. For the adjusted OR, the model adjusted for age, sex, marital status, educational level, occupation, monthly income, living situation, and distance of residence from the vaccination site

Figure 4 shows that compared to the standard-of-care group, the pay-it-forward participants reported higher confidence in the safety (*aOR* = 4.29; 95% *CI*, 1.78–11.50), importance (*aOR* = 5.15; 95% *CI*, 2.05–14.60), and effectiveness (*aOR* = 7.14; 95% *CI*, 2.36–27.50) of the vaccine, but there was no difference in the vaccine’s management (*aOR* = 3.06; 95% *CI* 1.02–10.80).

Overall, 54 of 104 pay-it-forward participants (51.9%, 54/104) contributed to the program, totaling US $105.11 and averaging US $1.95 per individual. The cost estimates and cost-effectiveness analyses are shown in Additional File 6 (Table S6). For the health provider, the total economic and financial costs of the pay-it-forward intervention were US $6983.57 and US $6878.46, corresponding to US $95.67 and US $94.23 per vaccinated person, respectively. In contrast, the standard-of-care group had a total cost of US $4178.37 or US $278.56 per vaccinated person. The incremental cost-effectiveness ratio for the pay-it-forward group compared to the standard-of-care group was an increase of US $48.37 in economic costs or US $46.55 in financial costs per additional person vaccinated.

### Qualitative findings

#### Demographic characteristics

Among the 13 interviewees, 53.8% were female, and 76.9% were married. The age range was from 63 to 81 years (median: 68 years), and most individuals had completed high school or higher education. All but one participant had received the vaccine. Additionally, 53.8% of participants reported donating (Additional file 7: Table S7).

### Donation acceptance and perceptions

The vast majority of participants interviewed expressed high acceptance of the donation concept, perceiving it as a reasonable and voluntary form of mutual assistance and social reciprocity. Primarily driven by altruism and a sense of gratitude after receiving the subsidized vaccination, most participants appreciated the noncoercive nature of the request and the respect shown to older adults. They strongly preferred the flexibility of giving within their means over mandatory or high fixed amounts. The primary reasons for refusal were financial constraints or concerns about transparency in donations. Participants also emphasized the importance of broader community outreach and a shared social consensus to enhance the program’s long-term sustainability (Additional file 7).

### Key factors associated with donation intentions

Qualitative analysis identified six major themes shaping participants’ willingness to donate: (1) Individual economic status, (2) altruism and social responsibility, (3) reciprocity after receiving benefits, (4) project perception and acceptance, and (5) trust in donation transparency (Additional file 7: Table S8).

## Discussion

This study demonstrated that the pay-it-forward intervention significantly increased the PPSV-23 uptake and boosted vaccine confidence among older adults. These findings suggest that the pay-it-forward strategy is beneficial for promoting the use of public healthcare services and enhancing community engagement and vaccine confidence among older adults with limited access to public health information due to economic, health, or social factors. Interestingly, the pay-it-forward intervention also led to higher influenza vaccination uptake, successful vaccine referrals, and a reduction in the per capita vaccination cost, generating substantial financial contributions to healthcare services. This strategy promotes vaccination through social interaction and mutual assistance, offering a more sustainable and scalable innovative approach to increasing vaccine uptake.

The results showed that participants in the pay-it-forward group had higher odds of receiving PPSV-23 vaccination compared to those in the standard-of-care group. This finding was consistent with our previous studies, which have shown that pay-it-forward interventions increased influenza vaccinations [30] and diagnostic testing for sexually transmitted infections among at-risk populations [22, 31]. The uptake of PPSV-23 (70.9%) was also higher than the coverage (30%–42%, 1%–24%) in other Chinese regions, where PPSV-23 was either free or partly reimbursed [39]. Moreover, the pay-it-forward strategy effectively promoted vaccination compared to other strategies, including free vaccinations [40], health education [14, 41], and community-based comprehensive intervention and Internet-based mechanisms [42]. The high effectiveness of the pay-it-forward strategy might be attributed to vaccine affordability support, increased community engagement, greater vaccine confidence, or a combination of these factors.

It is noteworthy that the pay-it-forward intervention generated a positive spillover effect, improving unsubsidized influenza vaccination rates. This outcome may be attributed to enhanced vaccine confidence, as participants demonstrated greater trust in vaccine safety, importance, and effectiveness. A similar mechanism was observed in our earlier study, where the intervention built trust in healthcare services through reciprocal generosity [22]. Vaccine confidence may mediate the relationship between the pay-it-forward intervention and vaccine uptake. Individuals are more likely to get vaccinated when they trust vaccines’ safety, efficacy, and necessity [43]. The intervention enhances older adults’ perceptions of the importance, effectiveness, and safety of vaccines, and this improved perception is associated with increased influenza vaccination uptake [44]. Furthermore, by emphasizing community solidarity through donations and postcards, the approach likely strengthened social cohesion—a key driver of health behavior in collectivist settings [45, 46]. By framing vaccination as a communal rather than individual responsibility, the pay-it-forward approach taps into intrinsic motivations for prosocial behavior, as predicted by upstream reciprocity theory [47].

Stratified analyses revealed more substantial pay-it-forward effects among urban residents, those with higher incomes, those who do not live alone, and individuals with chronic diseases. These findings are consistent with previous studies conducted in India [48], Europe [49], and China [12]. The disparity in intervention effects may stem from differences in the capacity to engage with the pay-it-forward model across socioeconomic groups. Higher socioeconomic status (e.g., urban residence, higher income) is associated with a greater propensity for altruistic behavior [50], which in turn makes individuals more likely to understand and accept pay-it-forward intervention. In addition, individuals with chronic diseases may place a higher value on vaccination due to a heightened perception of personal health risk [51]. Although limited by subgroup sample sizes, our findings preliminarily support pay it forward as a promising inclusive strategy, warranting larger studies to verify generalizability and optimize implementation across populations.

Experiments with humans show that gratitude, a positive emotion elicited by receiving something valuable, promotes prosocial behavior by encouraging direct and upstream reciprocity [47]. Our data suggest that the pay-it-forward intervention increased successful vaccine referrals, fostering a sense of gratitude among vaccinated individuals, which subsequently encouraged them to engage in prosocial behaviors, recommending the vaccine to others. However, successful vaccine referral is influenced by several factors, including knowledge and awareness of the vaccine among the recommended individuals, cultural and social dynamics, and the availability and cost of the vaccine [52]. Pay-it-forward interventions directly increase vaccination uptake and, through vaccine referral behaviors, indirectly expand community vaccination coverage, providing theoretical support and practical implications for vaccine promotion strategies.

The pay-it-forward intervention demonstrated superior outcomes despite higher total costs, achieving both increased vaccination coverage and reduced per-capita vaccination costs. This may be related to strengthening community engagement and fostering social solidarity through donations by leveraging intrinsic human tendencies for social connectedness and mutual support [53], as well as principles from upstream reciprocity theory [47]. First, the pay-it-forward intervention leverages the innate human desire for social connection by creating shared purpose in community settings, where proven strategies like community engagement boost vaccine coverage [54]. Recipients benefit directly from vaccination while empowered to support others through small donations or encouraging messages, establishing a self-sustaining cycle of mutual aid that increases participation at lower cost than conventional outreach. Second, drawing on upstream reciprocity theory, individuals who receive help are inclined to support others in turn [55]. The model initiates a ripple effect: each contribution encourages the next, extending the impact of the initial support. This builds vaccine confidence and uptake at the population level, decreases dependence on subsidies, reduces financial and administrative barriers, and strengthens community trust, ultimately improving public health outcomes.

This study received a lower donation rate and amount than in existing studies [30, 45]. This discrepancy may be due to the socioeconomic profile of our trial: over 60% of participants resided in rural areas with low income and low education levels. Some participants reported that their financial capacity was the primary factor determining the amount they donated (qualitative data). Empirical evidence consistently links higher socioeconomic status to greater pro-social behavior, including charitable donations, as wealthier individuals possess financial flexibility and exposure to altruistic norms [50]. A longitudinal study in India found that healthcare expenditures reduced household contributions to community health initiatives by 30–40% [56]. Older adults in our study faced competing financial demands related to managing chronic conditions, which likely constrained the disposable income available for donations.

Significantly, donation behavior was driven not solely by financial capacity but also significantly by nonmonetary factors, including altruism and social responsibility, a sense of reciprocity after benefiting from the project, and trust in the transparency of the donation management. The fact that over half of the participants donated, despite modest amounts, underscores the decisive role of these noneconomic motivators. This can be understood through the theory of generalized reciprocity, which posits that individuals who receive help are more likely to feel obligated to help others [57, 58], explaining the motivation to “give back.” Furthermore, participants’ trust in the transparency of donation management was a key facilitator. Existing research showed that institutional trust significantly predicted personal donations and volunteer activities [59], and transparency serves as a critical prerequisite for building such trust, enhancing donors’ commitment and thereby increasing their willingness to contribute [60]. Therefore, the lower monetary value should not be misinterpreted as low acceptability; rather, it reflects a context where nonfinancial motivations successfully spurred participation within the economic means of a community with limited disposable income.

While the pay-it-forward intervention showed positive effects in our study, its external validity to other settings must be cautiously evaluated. Key barriers to generalization include the following: Firstly, sustainability in resource-limited regions is challenging without external funding. Secondly, the approach is more feasible for lower-cost health services; high costs may limit the impact of donations. Thirdly, sociocultural differences in the acceptance of prosocial behaviors, such as “pay itforward,” could affect participation. Lastly, the level of preexisting community trust in institutions and the perceived transparency of the donation process are crucial to the intervention’s success.

Our study has several limitations. First, the lack of a community-based cluster randomized design posed a risk of contamination between the two groups. If participants in the control group were aware of the pay-it-forward intervention, they may have felt discouraged by the absence of a subsidy, potentially reducing their willingness to be vaccinated. This could have led to an overestimation of the intervention effectiveness. To mitigate this risk, participants were instructed not to share details of the intervention, tailored vaccination materials were provided to each group, and discussions were conducted in separate, designated spaces to ensure confidentiality and maintain study integrity. We assessed the potential for contamination through a sensitivity analysis, which showed that the intervention effect remained robust under different hypothetical scenarios (Additional file 8). Second, the cost-effectiveness analysis in this study focused only on short-term benefits, excluding disability-adjusted life years (DALYs) or quality-adjusted life years (QALYs) gained through vaccination, potentially underestimating the intervention’s long-term impacts. Incorporating DALYs and QALYs into cost-effectiveness analyses would provide a more comprehensive understanding of the pay-it-forward intervention’s impact on morbidity and mortality, as well as on overall quality of life. Furthermore, the current cost-effectiveness analysis does not account for several potential costs due to its narrow perspective. These omitted costs include patient-borne expenses, such as transportation and lost wages, as well as institutional operational overheads and critical vaccine-related indirect expenditures, such as cold chain maintenance and electricity consumption. Third, the interpretation of our stratified analyses is limited by small sample sizes in specific subgroups, leading to wide confidence intervals. Despite using Firth’s penalized-likelihood logistic regression to reduce bias, these estimates remain imprecise. The findings should therefore be seen as generating hypotheses about differential effects rather than providing conclusive evidence. Fourth, participants were recruited primarily through on-site community health centers, community networks, and social media (WeChat). This recruitment approach may have introduced selection bias, as our sample likely underrepresents marginalized older adults (e.g., those who are socially isolated, have limited mobility, or lack access to digital media), potentially limiting the generalizability of our findings to these underserved subgroups. Although the findings may not be fully generalizable to all older adults in China, especially those who seldom visit CHSCs, this study offers valuable experimental data that fills a critical gap in the existing literature. In future studies, we will employ strategies such as community outreach, referrals from family doctors, and multichannel promotion to more effectively engage these underserved subgroups. Fifth, operational differences between interventions prevented a strict double-blind design, making group allocation visible to researchers and participants. Finally, while some questionnaire items (e.g., vaccine confidence) may be subject to self-report bias, this did not affect the accuracy of vaccination coverage, which was verified through the vaccination information management system at the community health centers.

## Conclusions

In conclusion, the community-led pay-it-forward intervention significantly increased PPSV-23 vaccination uptake among older adults by addressing financial barriers and fostering community trust. Its scalability positions it as a viable strategy for advancing vaccine equity in low-resource settings, particularly where PPSV-23 vaccines are not fully subsidized. As global efforts to combat antimicrobial resistance and aging-related morbidity intensify, pay-it-forward offers a replicable model to bridge gaps in preventive care, aligning with the WHO’s 2030 Immunization Agenda. Future implementation science research could focus on integrating the pay-it-forward intervention into healthcare service subsidy policies. This may enhance social engagement and improve the accessibility, acceptability, and utilization of healthcare services within the community.

## Supplementary Information

Additional file 1. Baseline questionnaire.

Additional file 2. Follow-up questionnaire.

Additional file 3. Pneumococcal polysaccharide vaccination leaflet (23-valent pneumococcal polysaccharide vaccine).

Additional file 4. Pay-it-forward program leaflet.

Additional file 5. Table S1: Socio-demographic characteristics. Table S2: Multivariate logistic regression of PPSV-23 vaccination uptake (*n* = 214). Table S3: Multivariate logistic regression of influenza vaccination uptake (*n* = 214). Figure S1: Multivariate logistic regression of PPSV-23 vaccination uptake (stratified analysis, *n* = 214). Table S4: Successful pneumococcal vaccination referral to others (*n* = 214).

Additional file 6. Table S5: Unit cost (in 2023 USD) and frequency of vaccine use. Table S6: Cost-effectiveness analysis. Figure S2: Decision tree model. Figure S3: Univariate sensitivity analysis. Figure S3: Cost-effectiveness acceptability curve.

Additional file 7. Table S7: Demographic characteristics of interview participants. Table S8: Key themes and representative quotes on Factors influencing donation intentions.

Additional file 8. Odds ratios under hypothetical contamination scenarios.

## Data Availability

The datasets used and/or analysed during the current study are available from the corresponding author B.L. upon reasonable request.

## Abbreviations

aOR: Adjusted odds ratio
CAP: Community-acquired pneumonia
CHSC: Community health service centers
CHW: Community health workers
CI: Confidence interval
cOR: Crude odds ratio
PD: Pneumococcal disease
PPSV-23: 23-Valent pneumococcal polysaccharide vaccine
Spn: *S. pneumoniae*
WHO: World Health Organization
