# Supplementary material for: Pay-it-forward intervention increased pneumococcal vaccine uptake among older adults in China: a randomized controlled trial
Source: BMC Med. 2026 Jan 19;24:93. doi: 10.1186/s12916-026-04624-2 (PMC12895929; doi:10.1186/s12916-026-04624-2)
Supplement: Supplementary file 1 — Additional file 1. Baseline questionnaire. [file 12916_2026_4624_MOESM1_ESM.docx]

**Baseline questionnaire**

**Envelope number: Your name: Your ID number:**

**7. Your current living situation.**

A. Living with spouse

B. Living with someone not your spouse

C. Living alone

**8. Distance from your current residence to the vaccination site.**

A. Within 1 kilometer

B. [1-3) kilometers

C. [3-5) kilometers

D. More than 5 kilometers

**9. Have you smoked in the last six months?**

A. Regularly

B. Occasional

C. Never smoked

**10. Have you consumed alcohol in the last six months?**

A. Regularly

B. Occasional

C. Never smoked

**11. In the past year, have you ever suffered from any of the following diseases?** (Multiple choice)

A. Metabolic diseases: hypertension, hyperglycemia, hyperlipidemia, etc.

B. Digestive system diseases: chronic gastritis, chronic liver disease, stomach cancer, etc.

C. Skeletal system diseases: osteoarthritis, osteomalacia, osteoporosis, etc.

D. Respiratory system diseases: pneumonia, chronic bronchitis, bronchiectasis, chronic obstructive pulmonary disease, lung cancer, etc.

E. Cardiovascular system diseases: cerebral infarction, coronary heart disease, angina pectoris, acute myocardial infarction, congenital heart disease, rheumatic heart disease, etc.)

F. None of the above

**Part 1. Socio-demographic information**

**1. Your gender**：

A. Male

B. Female

**2. Your current legal marriage status is：**

A. Unmarried (living alone)

B. Unmarried (cohabiting)

C. Married

D. Divorced or widowed

**3. Your highest educational level is:**

A. Graduate students and above

B. University undergraduate

C. College

D. High school

E. Junior high school

F. Elementary school

**4. Your current occupation is：**

A. Company staff (white collar or office work)

B. Businessman/self-employed

C. Urban laborer (including migrant workers)

D. Unemployed/housewife/househusband

E. Teachers/Civil servants

/Enterprise workers/Healthcare workers

F. Farmer (working in rural areas)

G. Retired

H. Freelance

**5. Your current income per month:**

A. No income

B. (0,2000)RMB

C. [2000,3000) RMB

D. [3000,5000) RMB

E. [5000,10000) RMB

F. 10000 RMB or above

**6. Your place of residence in the last six months.**

A. City (city, county)

B. Township (market town, village)

**6. Older people with chronic illnesses are more likely to get pneumonia if they have not received the 23-valent pneumococcal polysaccharide vaccine.**

A. Yes

B. No

**7. Vaccination with 23-valent pneumococcal polysaccharide vaccine reduces hospitalizations and deaths.**

A. Yes

B. No

**8. Vaccination against 23-valent pneumococcal polysaccharide vaccine reduces medical costs due to pneumonia.**

A. Yes

B. No

**9. The 23-valent pneumococcal polysaccharide vaccine protects the old and the young for the same length of time.**

A. Yes

B. No

**Part 2. Knowledge of Pneumonia Vaccine**

**1. Have you known about pneumonia before?**

A. Yes

B. No

**2. Have you heard of the 23-valent pneumococcal polysaccharide vaccine before?**

A. Yes

B. No

**3. Which people do you think need the 23-valent pneumococcal polysaccharide vaccination the most? (Multiple choice)**

A. Children

B. Old adults

C. Immunocompromised or immunodeficient people

D. All people

E. Others _________________

**4. There is an age limit for vaccination with 23-valent pneumococcal polysaccharide vaccine.**

A. Yes

B. No

**5. Vaccination with 23-valent pneumococcal polysaccharide vaccine reduces the risk of pneumonia.**

A. Yes

B. No

**Part 3. Attitude toward Pneumonia Vaccine**

**1. It is beneficial to vaccinate with PPSV-23.**

A. Yes

B. No

**2. Many of my friends(older adults) have received the** **PPSV-23.**

A. Yes

B. No

C. Unclear

**3. Do you agree with the following statement?**

|  | Completely agree | Agree | Neutral | Disagree | Completely disagree |
| --- | --- | --- | --- | --- | --- |
| I think the 23-valent pneumococcal polysaccharide vaccine is effective in preventing pneumonia. | ○ | ○ | ○ | ○ | ○ |
| I trust the safety of the pneumonia  vaccine. | ○ | ○ | ○ | ○ | ○ |
| I have confidence in China’s strict vaccination management. | ○ | ○ | ○ | ○ | ○ |
| Overall, I think the 23-valent pneumococcal polysaccharide vaccine is important. | ○ | ○ | ○ | ○ | ○ |

**Part 4. Vaccine publicize and educate**

**1. You have been diagnosed with pneumonia.**

A. Yes

B. No (Skip to 3)

**2. You have been hospitalized for pneumonia.**

A. Yes

B. No

**3. You have been recommended by a healthcare professional to receive the 23-valent pneumococcal polysaccharide vaccine.**

A. Yes

B. No

**4. A relative or friend has recommended that you receive the 23-valent pneumococcal polysaccharide vaccine.**

A. Yes

B. No

**Part 5. Willingness of vaccination**

**1. Would you like to get vaccination of the 23-valent pneumococcal polysaccharide?**

A. Yes

B. No (Skip to 3)

**2. The reasons for you like to receive the 23-valent pneumococcal polysaccharide vaccine are (Multiple choice):**

A. Reduced risk of pneumonia and hospitalization

B. Vaccination recommended by a doctor (including those with underlying conditions such as immunocompromised and chronic diseases)

C. Vaccination recommended by family members or relatives

D. Reduce other illnesses caused by pneumonia

E. Affected by pneumonia due to COVID-19 infection

F. Others: _________________

**3. The reasons that make you hesitant or unwilling to receive the 23-valent pneumococcal polysaccharide vaccine are (Multiple choice):**

A. The price of vaccine is too expensive.

B. Streptococcus pneumoniae can also be infected after vaccination.

C. Adverse reactions may occur after vaccination.

D. I'm not susceptible to Streptococcus pneumoniae.

E. Vaccination sites are too far from home

F. I have received the COVID-19 vaccine and do not need the 23-valent pneumococcal polysaccharide vaccine.

G. I have a contraindication to vaccination.

H. Others: _________________

**4. Even if I catch pneumonia, I can get better quickly without medication or treatment.**

A. Yes

B. No

**5.** **Even if I catch pneumonia, it will not result in a heavy financial burden on the family.**

A. Yes

B. No

**6.** **Even if I catch pneumonia, it will not cause severe health problems.**

A. Yes

B. No

**7.** **Does vaccination with 23-valent pneumococcal polysaccharide vaccine reduce hospitalisations and deaths?**

A. Yes

B. No

**8.** **Do you think it takes a lot of time and effort to go for vaccinations?**

A. Yes

B. No

**9.** **You have a family member or friend who opposes you getting the 23-valent pneumococcal polysaccharide vaccine.**

A. Yes

B. No

**10. A healthcare professional is against you getting the 23-valent pneumococcal polysaccharide vaccine.**

A. Yes

B. No

**11.** **Have you ever hesitated to get the 23-valent pneumococcal polysaccharide vaccine? (other than for allergic reasons)**

A. Yes

B. No

**12.** **Have you ever refused to get the 23-valent pneumococcal polysaccharide vaccine? (including reasons for allergy)**

A. Yes

B. No

**Part 7. Pay-it-forward program (additional questions for participants in the pay-it-forward group)**

The last participant who participated in “pay it forward program” received the 23-valent pneumococcal polysaccharide vaccine and made a donation, he/she paid part of the cost of the pneumonia vaccine for you, you accepted his/her donation and used it to receive the 23-valent pneumococcal polysaccharide vaccine, and then you can choose to pass on the love, i.e., donate some money or a greeting card to support more people to receive the pneumonia vaccine.

**1. What do you think about the benefits of "** **pay it forward " for vaccination?**

A. Reduce vaccine costs

B. Benefit my health

C. Make more people get vaccinated

D. Spreading love and warmth

E. No benefit

F. Other:_________

**2. Acceptability of Intervention Measure (AIM)**

|  | Completely agree | Agree | Neutral | Disagree | Completely disagree |
| --- | --- | --- | --- | --- | --- |
| (Pay-it-forward program) meets my needs | ○ | ○ | ○ | ○ | ○ |
| (Pay-it-forward program) is appealing to me | ○ | ○ | ○ | ○ | ○ |
| I like (Pay-it-forward program) | ○ | ○ | ○ | ○ | ○ |
| I welcome (Pay-it-forward program) | ○ | ○ | ○ | ○ | ○ |

**3. Intervention Appropriateness Measure (IAM)**

|  | Completely agree | Agree | Neutral | Disagree | Completely disagree |
| --- | --- | --- | --- | --- | --- |
| (Pay-it-forward program) seems fitting | ○ | ○ | ○ | ○ | ○ |
| (Pay-it-forward program) seems suitable |  |  |  |  |  |
| (Pay-it-forward program) seems applicable | ○ | ○ | ○ | ○ | ○ |
| (Pay-it-forward program) seems like good match |  |  |  |  |  |

**4. Feasibility of Intervention Measure (FIM)**

|  | Completely agree | Agree | Neutral | Disagree | Completely disagree |
| --- | --- | --- | --- | --- | --- |
| (Pay-it-forward program) seems implementable | ○ | ○ | ○ | ○ | ○ |
| (Pay-it-forward program) seems possible | ○ | ○ | ○ | ○ | ○ |
| (Pay-it-forward program) seems doable |  |  |  |  |  |
| (Pay-it-forward program) seems easy to use | ○ | ○ | ○ | ○ | ○ |

**5. Would you like to donate some money to support the next family to receive the 23-valent pneumococcal polysaccharide vaccine?**

A. Yes

B. No

**6. How much would you like to donate to support the next vaccination participant?**

A. 200 RMB

B. 100 RMB

C. 50 RMB

D. 20 RMB

E. Other: _________________

**7. Your telephone number: ____________**
