# Supplementary material for: Pay-it-forward intervention increased pneumococcal vaccine uptake among older adults in China: a randomized controlled trial
Source: BMC Med. 2026 Jan 19;24:93. doi: 10.1186/s12916-026-04624-2 (PMC12895929; doi:10.1186/s12916-026-04624-2)
Supplement: Supplementary file 2 — Additional file 2. Follow-up questionnaire. [file 12916_2026_4624_MOESM2_ESM.docx]

**Follow-up questionnaire**

**ID Number:**

**Your name:**

**Part 1. Vaccination intentions and reasons**

**1. Are you currently vaccinated with the 23-valent pneumococcal polysaccharide vaccine?**

A. Yes (Skip to 3)

B. No

**2. The reason you did not receive the 23-valent pneumococcal polysaccharide vaccine**

|  | Yes | No |
| --- | --- | --- |
| I don't know about the 23-valent pneumococcal polysaccharide vaccine | ○ | ○ |
| I may have an adverse reaction after vaccination | ○ | ○ |
| I only get free vaccines | ○ | ○ |
| I think the vaccine is more expensive | ○ | ○ |
| I can get Streptococcus pneumoniae even after vaccination | ○ | ○ |
| I don't think I'm susceptible to Streptococcus pneumoniae | ○ | ○ |
| Short time to market for vaccines | ○ | ○ |
| I have received the COVID-19 vaccine, so I don't need the 23-valent pneumococcal polysaccharide vaccine. | ○ | ○ |
| I have a contraindication to vaccination. | ○ | ○ |
| I don't think vaccines are safe. | ○ | ○ |
| My family is against my inoculation. | ○ | ○ |
| Other: | ○ | ○ |

**3. Adverse events were reported two weeks after vaccination.**

|  | Yes | No |
| --- | --- | --- |
| **General reaction** |  |  |
| Fever (body temperature >37.3°C) | ○ | ○ |
| Injection site pain | ○ | ○ |
| Injection site pruritus ( > 2.5 cm) | ○ | ○ |
| Itching at the vaccination site | ○ | ○ |
| skin rash | ○ | ○ |
| Injection site sclerosis（＞2.5cm） | ○ | ○ |
| Nausea or vomiting | ○ | ○ |
| Headache | ○ | ○ |
| Fatigue or tiredness | ○ | ○ |
| muscle soreness | ○ | ○ |
| other | ○ | ○ |
| **Infections and infestations** | | |
| Pneumonia | ○ | ○ |
| Colds/Coughs | ○ | ○ |
| Bronchitis | ○ | ○ |
| Nasopharyngitis | ○ | ○ |
| Acute sinusitis | ○ | ○ |
| Meningitis pneumococcal | ○ | ○ |
| Gastroenteritis | ○ | ○ |
| others | ○ | ○ |

**Part 2. Knowledge of 23-valent Pneumococcal polysaccharide Vaccine**

**1. Have you known about pneumonia before?**

A. Yes

B. No

**2. Have you heard of the 23-valent pneumococcal polysaccharide vaccine before?**

A. Yes

B. No

**3. Which people do you think need the 23-valent pneumococcal polysaccharide vaccination the most? (Multiple choice)**

A. Children

B. Older adults

C. Immunocompromised or immunodeficient people

D. All people

E. Others _________________

**4. There is an age limit for vaccination with the 23-valent pneumococcal polysaccharide vaccine.**

A. Yes

B. No

**5. Vaccination with the 23-valent pneumococcal polysaccharide vaccine reduces the risk of pneumonia.**

A. Yes

B. No

**6. Older people with chronic illnesses are more likely to get pneumonia if they have not received the 23-valent pneumococcal polysaccharide vaccine.**

A. Yes

B. No

**7. Vaccination with the 23-valent pneumococcal polysaccharide vaccine reduces hospitalizations and deaths.**

A. Yes

B. No

**8. Vaccination against the 23-valent pneumococcal polysaccharide vaccine reduces medical costs due to pneumonia.**

A. Yes

B. No

**9. The 23-valent pneumococcal polysaccharide vaccine protects the old and the young for the same length of time.**

A. Yes

B. No

**Part 3. Attitude of the 23-valent pneumococcal polysaccharide**

**1. Vaccination with the 23-valent pneumococcal polysaccharide vaccine is beneficial.**

A. Yes

B. No

**2. Does vaccination with the 23-valent pneumococcal polysaccharide vaccine reduce hospitalisations and deaths?**

A. Yes

B. No

**3. Even if I catch pneumonia, I can get better quickly without medication or treatment.**

A. Yes

B. No

**4. Even if I catch pneumonia, it will not result in a heavy financial burden on the family.**

A. Yes

B. No

**5. Even if I catch pneumonia, it will not cause severe health problems.**

A. Yes

B. No

**6. Do you think it takes a lot of time and effort to go for vaccinations?**

A. Yes

B. No

**7. Do you agree with the following:**

|  | Completely agree | Agree | Neutral | Disagree | Completely disagree |
| --- | --- | --- | --- | --- | --- |
| I think the 23-valent pneumococcal polysaccharide vaccine is effective in preventing pneumonia. | ○ | ○ | ○ | ○ | ○ |
| I trust the safety of the 23-valent pneumococcal polysaccharide  vaccine. | ○ | ○ | ○ | ○ | ○ |
| I believe China’s strict vaccination  management. | ○ | ○ | ○ | ○ | ○ |
| Overall, I think the 23-valent pneumococcal polysaccharide vaccine is important. | ○ | ○ | ○ | ○ | ○ |

**Part 4. Pay-it-forward program (additional questions for participants in the pay-it-forward group)**

The last participant who participated in “Pay-it-forward study” received the 23-valent pneumococcal polysaccharide vaccine and made a donation, he/she paid part of the cost of the pneumonia vaccine for you, and you accepted his/her donation and used it to receive the 23-valent pneumococcal polysaccharide vaccine, and then you can choose to pass on the love, i.e., donate some money or a greeting card to support more people to receive the pneumonia vaccine.

**1. What reasons do you think may be associated with the advocacy of the pay-it-forward strategy (Multiple choice)?**

A. Not enough money donated to make it sustainable

B. Not enough publicity

C. It takes a long time

D. Insufficient funds

E. People's participation is not high

F. Other: _______________

**2. What factors do you think might motivate people to donate? (Multiple choice)**

A. Higher income

B. Family support

C. Feeling that the study is meaningful

D. They are caring and socially responsible

E. They benefit from the study

F. They want to encourage others to get vaccinated

G. Others: _________________

**3. What factors do you think might discourage people from donating? (Multiple choice)**

A. Low income

B. Family members are not supportive

C. They don't find the study meaningful

D. Doubt about where the donation will go

E. They don't benefit from the study

F. They just don't want to donate

G. Other: _________________

**Part 5. Vaccine recommendation**

**1. Did you get an influenza vaccine after participating in the program**

A. Yes

B. No

**2. Have you successfully recommended others to get the 23-valent pneumococcal polysaccharide vaccine since participating in the program?**

A. Yes

B. No (Skip to 5)

**3. In which ways did you recommend the vaccine to them? [Multiple choice]**

A. At family or friend gatherings, such as Chinese square fitness dancing, chatting with neighbors or friends

B. By phone calls or text messages

C. Face-to-face recommendations

D. Social media (e.g., WeChat, TikTok, etc.)

E. Other: ________________

**4.** **Whom did you successfully recommend the vaccine to? Please provide their name, gender, approximate age, and phone number so we can verify in the vaccination system.**

| Name | Gender | Age | Phone Number |
| --- | --- | --- | --- |
|  |  |  |  |
|  |  |  |  |
|  |  |  |  |
|  |  |  |  |

**5. Your phone number:________________**
