# Supplementary material for: Pay-it-forward intervention increased pneumococcal vaccine uptake among older adults in China: a randomized controlled trial
Source: BMC Med. 2026 Jan 19;24:93. doi: 10.1186/s12916-026-04624-2 (PMC12895929; doi:10.1186/s12916-026-04624-2)
Supplement: Supplementary file 3 — Additional file 3. Pneumococcal polysaccharide vaccination leaflet (23-valent pneumococcal polysaccharide vaccine). [file 12916_2026_4624_MOESM3_ESM.pdf]

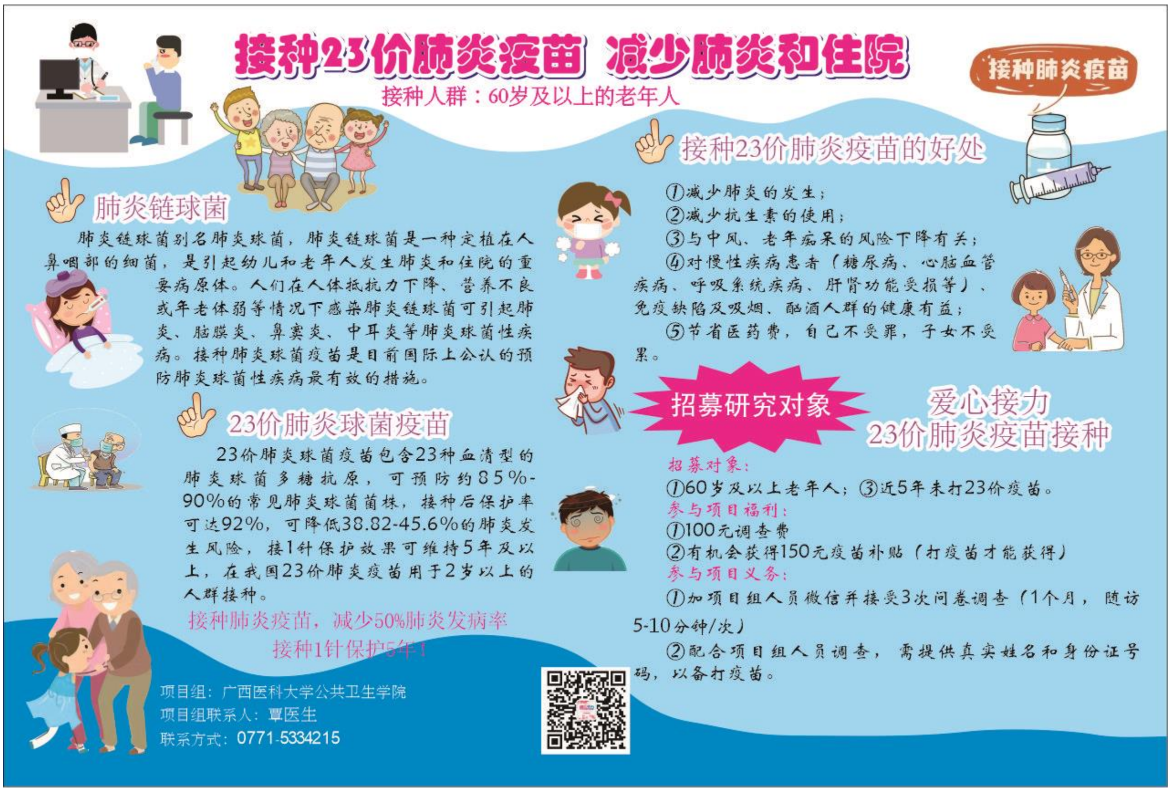

23-valent pneumococcal polysaccharide vaccination to reduce pneumonia and hospitalization rates

Vaccination Population: People aged 60 years and older

Streptococcus pneumoniae

Streptococcus pneumoniae, called pneumococcus, is a bacterium that colonizes the nasopharynx in humans and is an important pathogen that causes pneumonia and hospitalization in old adults. When people are infected with streptococcus pneumoniae under conditions such as decreased resistance, malnutrition or old age, it can cause pneumococcal diseases such as pneumonia, meningitis, sinusitis and otitis media. Pneumococcal vaccination is currently recognized as the most effective preventive measure against pneumococcal diseases worldwide.

23-valent pneumococcal polysaccharide vaccine

The 23-valent pneumococcal polysaccharide vaccine contains 23 serotypes of pneumococcal polysaccharide antigens and protects against about 85-90% of common pneumococcal strains. After vaccination, the protection rate can reach 92%, which can reduce the risk of pneumonia by 38.82-45.6%. The protection effect of one dose can be maintained at least 5 years, and the 23-valent pneumococcal polysaccharide vaccine is used for people over 2 years old in China.

Benefits of vaccination with 23-valent pneumococcal polysaccharide vaccine

① Reduce the incidence of pneumonia;

② Reduce the use of antibiotics;

③Associated with a decrease of the risk of stroke and Alzheimer's disease;

④ It is benefit to the health of people with chronic diseases (diabetes, cardiovascular and cerebrovascular diseases, respiratory diseases, impaired liver and kidney functions, etc.), immunodeficiency, smoking, and excessive drinking;

⑤ It saves medical expenses, does not make you suffer, and does not burden your children.

Recruitment of participants

①People aged 60 years and older; ②who have not got the 23-valent pneumococcal polysaccharide vaccine in the last 5 years.

Benefits of participating in the program

① 100 RMB survey fee.

② Opportunity to obtain 150 RMB vaccine subsidy (only for vaccination).

Duty of participating in the study

① Add WeChat of the research team and agree to receive three questionnaires survey(one month, 5-10 minute/time)

② Coordinate with Team Member for the investigation, provide your real name and ID number for vaccination.
