# Supplementary material for: Pay-it-forward intervention increased pneumococcal vaccine uptake among older adults in China: a randomized controlled trial
Source: BMC Med. 2026 Jan 19;24:93. doi: 10.1186/s12916-026-04624-2 (PMC12895929; doi:10.1186/s12916-026-04624-2)
Supplement: Supplementary file 5 — Additional file 5. Table S1: Socio-demographic characteristics. Table S2: Multivariate logistic regression of PPSV-23 vaccination uptake (n = 214). Table S3: Multivariate logistic regression of influenza vaccination uptake (n = 214). Figure S1: Multivariate logistic regression of PPSV-23 vaccination uptake (stratified analysis, n = 214). Table S4: Successful pneumococcal vaccination referral to others (n = 214). [file 12916_2026_4624_MOESM5_ESM.pdf]

Per-protocol analyses

Table S1. Socio-demographic characteristics of recruited participants aged ≥60 years in Nanning City, China (n=214)

Variables	All (n=214)	Standard-of-care (n=110)	Pay-it-forward (n=104)	*P value*
**Sex**				
Male	91 (42.5)	45 (40.9)	46 (44.2)	0.724
Female	123 (57.5)	65 (59.1)	58 (55.8)	
**Age (Year)**				
60-65	63 (29.4)	34 (30.9)	29 (27.9)	0.339
66-70	64 (29.9)	28 (25.5)	36 (34.6)	
≥71	87 (40.7)	48 (43.6)	39 (37.5)	
**Marital status**				
Married	175 (81.8)	85 (77.3)	90 (86.5)	0.115
Unmarried/Divorced/widowed	39 (18.2)	25 (22.7)	14 (13.5)	
**Occupation**				
Farmer	33 (15.4)	22 (20)	11 (10.6)	0.162
Retired	156 (72.9)	76 (69.1)	80 (76.9)	
Others	25 (11.7)	12 (10.9)	13 (12.5)	
**Monthly income (US$)**				
[0,276)	64 (29.9)	41 (37.3)	23 (22.1)	0.015
[276,414)	56 (26.2)	30 (27.3)	26 (25)	
[414,690)	46 (21.5)	23 (20.9)	23 (22.1)	
≥690	48 (22.4)	16 (14.5)	32 (30.8)	
**Living status**				
Living with spouse	149 (69.6)	68 (61.8)	81 (77.9)	0.016
Living without spouses	65 (30.4)	42 (38.2)	23 (22.1)	
**Area of residence**				
Urban	99 (46.3)	47 (42.7)	52 (50)	0.353
Rural	115 (53.7)	63 (57.3)	52 (50)	
**Distance of residence from the vaccination site**				
Within 1km	76 (35.5)	34 (30.9)	42 (40.4)	0.278
[1-3) km	69 (32.2)	36 (32.7)	33 (31.7)	
≥3km	69 (32.2)	40 (36.4)	29 (27.9)	
**Smoking in the last 6 months**				
No	182 (85)	96 (87.3)	86 (82.7)	0.455
Yes	32 (15)	14 (12.7)	18 (17.3)	
**Drinking in the last 6 months**				
No	157 (73.4)	82 (74.5)	75 (72.1)	0.805

Yes	57 (26.6)	28 (25.5)	29 (27.9)	
**Chronic disease**				
No	67 (31.3)	38 (34.5)	29 (27.9)	0.367
Yes	147 (68.7)	72 (65.5)	75 (72.1)	
**Educational level**				
High school and above	98 (45.8)	55 (50)	43 (41.3)	0.257
Middle school and below	116 (54.2)	55 (50)	61 (58.7)	

Table S2. Multivariate logistic regression of PPSV-23 vaccination uptake in two groups (n = 214)

Group	Pneumococcal vaccine		
	**Vaccination n(%)**	**cOR (95 CI)**	**aOR (95 CI)**
**Standard-of-care**	15(13.6)	1	1
**Pay-it-forward**	73(70.2)	14.90 (7.68, 30.60)	16.30 (7.94, 35.80)
***P* value**		$p < 0.001$	$p < 0.001$

For adjusted OR, the model adjusted for age, sex, area of residence, marital status, educational level, occupation, monthly income, living situation, and distance of residence from vaccination site.

Table S3 Multivariate logistic regression of influenza vaccination uptake in two groups (n = 214)

Group	Influenza vaccine		
	**Vaccination n(%)**	**cOR (95 CI)**	**aOR (95 CI)**
**Standard-of-care**	19 (17.3)	1	1
**Pay-it-forward**	33 (31.7)	2.23 (1.18, 4.30)	2.42 (1.23, 4.91)
***P* value**		$p = 0.015$	$p = 0.012$

For adjusted OR, the model adjusted for age, sex, area of residence, marital status, educational level, occupation, monthly income, living situation, and distance of residence from vaccination site.

Subgroup	Intervention	Crude Odds Ratio	cOR (95%CI)	*P*
**Monthly income**				
Lower income (N = 120)	PIF		7.13 (3.14, 17.10)	< 0.001
Higher income (N = 94)	PIF		48.00 (14.20, 227.00)	< 0.001
**Living status**				
Living with spouses (N = 149)	PIF		14.80 (6.78, 34.90)	< 0.001
Living without spouses (N = 65)	PIF		12.30 (3.55, 52.10)	< 0.001
**Chronic disease or not**				
With Chronic disease (N = 147)	PIF		15.90 (7.16, 38.60)	< 0.001
Without Chronic disease (N = 67)	PIF		12.50 (3.98, 46.30)	< 0.001
**Area of residence**				
Urban (N = 99)	PIF		28.70 (10.20, 93.90)	< 0.001
Rural (N = 115)	PIF		8.86 (3.74, 22.80)	< 0.001

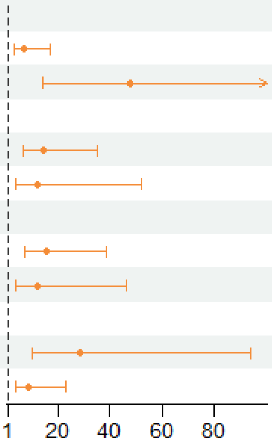

Subgroup	Intervention	Ajusted Odds Ratio	aOR (95%CI)	*P*
**Monthly income***				
Lower income (N = 120)	PIF		6.20 (2.72, 15.20)	< 0.001
Higher income (N = 94)	PIF		35.50(9.89, 189)	< 0.001
**Living status****				
Living with spouses (N = 149)	PIF		12.40 (5.66, 29.90)	< 0.001
Living without spouses (N = 65)	PIF		9.06 (2.64, 38.40)	< 0.001
**Chronic disease or not †**				
With Chronic disease (N = 147)	PIF		11.70(5.22, 28.60)	< 0.001
Without Chronic disease (N = 67)	PIF		10.90(3.37, 45.10)	< 0.001
**Area of residence ††**				
Urban (N = 99)	PIF		28.30(8.71, 127.00)	< 0.001
Rural (N = 115)	PIF		7.66(3.16, 20.60)	< 0.001

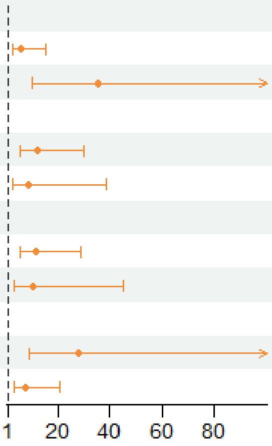

Figure S1. Multivariate logistic regression of PPSV-23 vaccination uptake in the two groups (stratified analysis, n=214).

Note: PIF: Pay-it-forward. Reference: Standard-of-care. *For adjusted OR, the model adjusted for age, sex, area of residence, marital status, educational level, occupation, living situation, and distance of residence from the vaccination site. **For adjusted OR, the model adjusted for age, sex, area of residence, educational level, occupation, monthly income, and distance of residence from the vaccination site. †For adjusted OR, the model adjusted for age, sex, marital status, area of residence, educational level, occupation, monthly income, living situation, and distance

of residence from the vaccination site. ††For adjusted OR, the model adjusted for age, sex, marital status, educational level, occupation, monthly income, living situation, and distance of residence from the vaccination site.

Table S4 Successful pneumococcal vaccination referral to others (n=214)

**Group**	**Total**	**PPSV-23 referral success**		***P value***
		**No**	**Yes**	
**Standard-of-care**	110(51.4)	98 (89.1)	12 (10.9)	0.336
**Pay-it-forward**	104(48.6)	87 (83.7)	17 (16.3)	
