# Supplementary material for: Pay-it-forward intervention increased pneumococcal vaccine uptake among older adults in China: a randomized controlled trial
Source: BMC Med. 2026 Jan 19;24:93. doi: 10.1186/s12916-026-04624-2 (PMC12895929; doi:10.1186/s12916-026-04624-2)
Supplement: Supplementary file 6 — Additional file 6. Table S5: Unit cost (in 2023 USD) and frequency of vaccine use. Table S6: Cost-effectiveness analysis. Figure S2: Decision tree model. Figure S3: Univariate sensitivity analysis. Figure S3: Cost-effectiveness acceptability curve. [file 12916_2026_4624_MOESM6_ESM.pdf]

Table S5 Unit cost (in 2023 USD) and frequency of vaccine use in pay-it-forward and standard-of-care groups

Intervention	Cost items	Unit cost(USD)	Resource use	Source
Pay-it-forward		Staff wage per hour (USD/hour)*		
Start-up costs				
	Time spent by fellows preparing for workshops†	10.22	1*5h	Personal communication with research staff
	Time spent by research assistant preparing for workshops†	6.81	1*5h	Personal communication with research staff
	Cost of nurse's time (participation in discussion of PIF program)	5.45	4*1h	Guangxi Talent Network Salary Report 2023[1]
	Cost of clinic coordinators(participation in discussion of the PIF program)	5.45	4*1h	Guangxi Talent Network Salary Report 2023[1]
Recurrent costs				
	Cost of per vaccine	21.29	73	Health clinic reimbursement invoices
	Cost of time for the nurse to recruit participants	5.45	4*40h	Guangxi Talent Network Salary Report 2023[1]
	Cost of time for the nurse to follow up with participants	5.45	4*50h	Guangxi Talent Network Salary Report 2023[1]
	Time cost of clinic coordinators in performing administrative work for the PIF program	5.45	4*40h	Guangxi Talent Network Salary Report 2023[1]
		Cost per item / batch (USD)		
	Leaflet design costs	21.29	1	Project research staff invoices
	Cost of printing leaflets(batch cost)	69.18	104	Project research staff invoices
	Cost of envelopes(batch cost)	3.55	250	Project research staff invoices
	Cost of postcards(batch cost)	3.12	1	Project research staff invoices
Fixed costs	Cost of hiring vaccinators for the four clinics	5.45	4*70h	Guangxi Talent Network Salary Report 2023[1]
Standard-of-care		Staff wage per hour (USD/hour)*		
Start-up costs				
	Cost of nurse’s time(participation in discussion of PIF program)	5.45	4*1h	Guangxi Talent Network Salary Report 2023[1]
	Cost of clinic coordinators(participation in discussion of PIF program)	5.45	4*1h	Guangxi Talent Network Salary Report 2023[1]
Recurrent costs				
	Cost of per vaccine	21.29	15	Health clinic reimbursement invoices
	Cost of time for the nurse to recruit participants to receive PPSV-23 vaccination	5.45	4*20h	Guangxi Talent Network Salary Report 2023[1]
	Cost of time for the nurse to follow up with participants	5.45	4*50h	Guangxi Talent Network Salary Report 2023[1]
	Time cost of clinic coordinators in performing administrative work for PPSV-23 vaccination	5.45	4*20h	Guangxi Talent Network Salary Report 2023[1]
		Cost per item / batch (USD)		
	Leaflet design costs	7.1	1	Project research staff invoices
	Cost of printing leaflets(batch cost)	23.06	1	Project research staff invoices
	Cost of envelopes(batch cost)	3.55	250	Project research staff invoices
Fixed costs				
	Cost of hiring vaccinators for the four clinics	5.45	4*70h	Guangxi Talent Network Salary Report 2023[1]

Note: PIF - pay-it-forward, USD – United States dollars.

*Hourly wages were calculated from monthly or yearly wages based on an assumption of 250 working days per year and 8 working hours per day.

†The costs were annualized over a three-year period at a 3% discount rate, as the training, materials, and expertise gained through these sessions are expected to continue providing value over the next three years. These resources will inform future iterations of the pay-it-forward vaccination intervention program and were therefore annualized over three years.

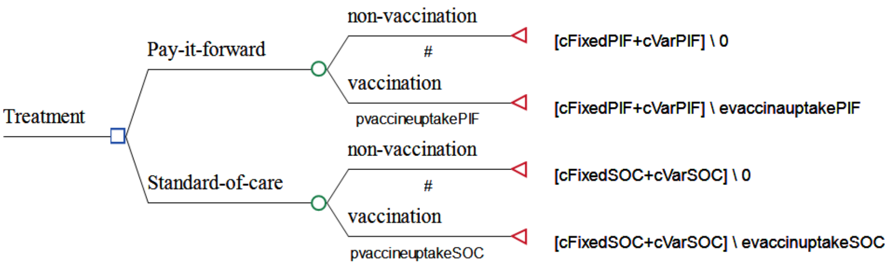

Figure S2. Decision tree model.

Table S6 Cost-effectiveness analysis of the standard-of-care and pay-it-forward groups

	Pay-it-forward	Standard-of-care
Economic cost(USD)	6983.57	4178.37
Donation contributions (USD)	105.11	-
Financial cost(USD)	6878.46	4178.37
PPSV-23 uptake	73	15
Average cost of vaccination per person(USD)	95.67	278.56
ICER(Economic cost)	48.37	-
ICER(Financial cost)	46.55	-

Note: ICER: incremental cost-effectiveness ratio.

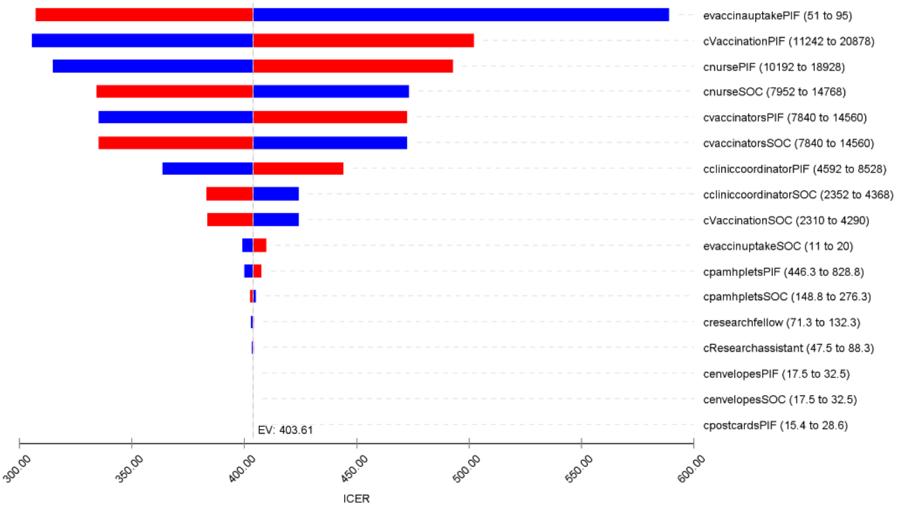

Figure S3. Univariate sensitivity analysis compared to pay-it-forward vs. standard-of-care cost-effectiveness (ICER of economic cost per person vaccinated).

Note: Costs are in Renminbi (RMB). PIF: pay-it-forward; SOC: Standard-of-care; evaccinauptakePIF: Pay-it-forward group vaccination numbers. cVaccinationPIF: Pay-it-forward group vaccination costs. cnursePIF: Time cost of nurses in the Pay-it-forward group. cnurseSOC: Time cost of nurses in the standard-of-care group. cvaccinatorsPIF: The cost of hiring vaccinators for the pay-it-forward group. cvaccinatorsSOC: The cost of hiring vaccinators for the standard-of-care group. ccliniccoordinatorPIF: Time cost of clinic coordinators in the pay-it-forward group. ccliniccoordinatorSOC: Time cost of clinic coordinators in the standard-of-care group. cVaccinationSOC: Standard-of-care group vaccination costs. evaccinuptakeSOC: Standard-of-care group vaccination numbers. cpamhpletsPIF: The cost of leaflet for the Pay-it-forward group. cpamhpletsSOC: The cost of leaflet for the standard-of-care. cresearchfellow: The time cost for research fellows to conduct workshops for conceptualizing the Pay-it-forward program. cResearchassistant: The time cost for research assistants participating in the Pay-it-forward program workshops. cenvelopesPIF: The cost of envelopes for the pay-it-forward group. cenvelopesSOC: The cost of envelopes for the standard-of-care group. cpostcardsPIF: The cost of postcards for the Pay-it-forward group.

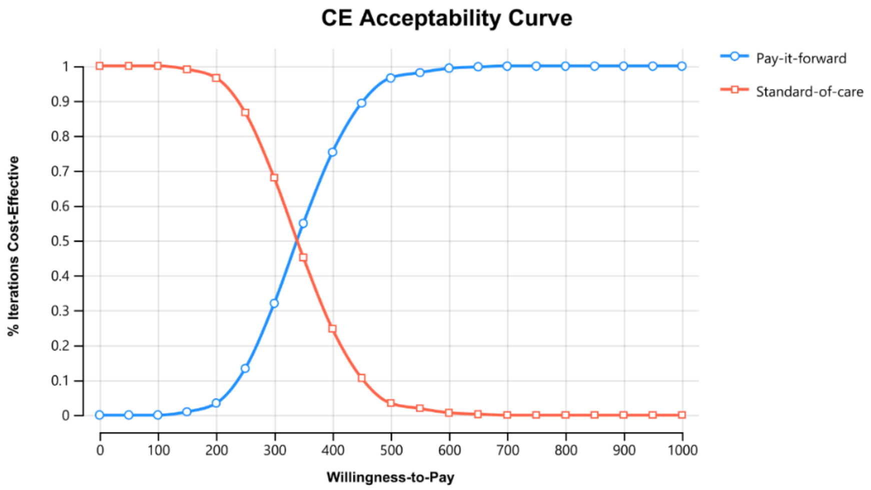

Figure S4. Cost-effectiveness acceptability curve (economic cost).

Note: Costs are in Renminbi (RMB).
