# Supplementary material for: Pay-it-forward intervention increased pneumococcal vaccine uptake among older adults in China: a randomized controlled trial
Source: BMC Med. 2026 Jan 19;24:93. doi: 10.1186/s12916-026-04624-2 (PMC12895929; doi:10.1186/s12916-026-04624-2)
Supplement: Supplementary file 7 — Additional file 7. Table S7: Demographic characteristics of interview participants. Table S8: Key themes and representative quotes on Factors influencing donation intentions. [file 12916_2026_4624_MOESM7_ESM.pdf]

Qualitative findings

I. Demographic Characteristics of Interviewees

Table S7. Demographic Characteristics of Interview Participants

Number	Sex	Age (years old)	Occupation	Education	Marital Status	Ethnicity	Vaccination Status	Donation Status
01	Male	76	Retired	College	Married	Han	Vaccinated	Donated
02	Female	71	Retired	Technical secondary school	Widowed	Han	Vaccinated	Donated
03	Female	68	Retired	Senior high school	Married	Han	Vaccinated	Donated
04	Female	81	Retired	Senior high school	Married	Han	Vaccinated	Donated
05	Female	67	Retired	Senior high school	Married	Han	Vaccinated	Donated
06	Female	74	Retired	Senior high school	Widowed	Zhuang	Vaccinated	No donation
07	Male	68	Retired	Senior high school	Married	Han	Vaccinated	Donated
08	Male	70	Retired	College	Married	Han	Vaccinated	No donations
09	Female	70	Retired	College	Married	Man	Vaccinated	Donated
10	Male	65	Retired	College	Married	Han	Vaccinated	No donation
11	Male	68	Retired	Primary school	Married	Zhuang	Vaccinated	No donation
12	Female	63	Retired	Senior high school	Widowed	Zhuang	Vaccinated	No donation
13	Male	64	Retired	College	Married	Han	Not vaccinated	No donation

II. Donation Acceptance, Perceptions

Overall, acceptance of the donation concept was high, with most participants considering it an acceptable and reasonable practice that emphasizes the principle of giving within one's means. Several interviewees viewed donating as a form of mutual assistance and a way to give back to society. Their participation was primarily driven by altruism and a willingness to contribute after receiving benefits.

"There's no particular issue — I just accept it. I feel it's a way of passing on an act of kindness." -02

"Go ahead and donate. If someone else has a problem, the money can be used. It's

quite acceptable." -04

"I think it's a good thing and still acceptable." -09

"It's legitimate and acceptable. It benefits the people, contributes to society, and helps others." -10

"It's appropriate. Being able to help others is the best thing." -12

A minority of participants expressed confusion or refusal, citing a lack of trust in the project or the belief that fundraising should not target the elderly. Those who declined to donate were primarily motivated by financial constraints or trust concerns.

"I'm not willing. I would never take part in donating money or scanning a QR code. There's nothing I want, and if I'm asked to give money, I would definitely refuse." -06

"I don't think it's good. The purpose of getting vaccinated is to get early prevention and avoid the illness. This project should be initiated by the state, not require elderly patients to donate money. I don't think that's a good approach." -06

"Anyway, I'm a bit confused; I don't understand why this step (donation) exists." -07

III. Views on the project and donation design

Participants generally perceived the donation request process as natural and non-coercive, and they appreciated the respect afforded to older adults by the staff.

One participant stated, *"There was no pressure; it was all voluntary. He (the staff) asked me about donating, and I said I'd think about it first. We just consider it ourselves." -05*

Another shared, *"There was no coercion. They just said that if we have the ability, we could help others. They had a very good attitude." - 12*

Similarly, one participant explained, *"There was no pressure. The people around me are all very kind, and they respect us older folks. They didn't force anything. So when they invited me, I was happy to come." -13*

Most participants preferred to donate according to their personal choice, rejecting mandatory contributions or disproportionately high fixed amounts. A minority, however, supported establishing a reasonable donation range as a guidance.

"I like it best when we can donate whatever we want. I don't see the point of setting an amount — five or ten yuan isn't much anyway. I give according to my own ability and wishes, and however I choose to contribute is an expression of love." -01

"I think it should be whatever you want to give. For me, donating is really about giving according to your own means." -07

"It might be better to have a set amount, because some people might say they'll only give one yuan or just a small amount. You need a minimum or maximum standard." -03

"Having a standard is a bit better — it helps you see how much to give. If you give too little, it feels awkward; if you give too much, you might not have enough." -04

Participants generally recommended enhancing outreach and fostering a shared social consensus, reflecting a heightened concern for the programme's long-term sustainability.

"Your promotion isn't really enough. I came because the staff notified me, and when I arrived, many of my friends didn't even know about it. You could put up an advert — we dance outside every day, you could go to where we dance and hand out flyers." -03

"You need to mobilise older people and promote it."-09

"On the basis of wide promotion, society can reach a consensus and reduce the government's pressure. Let the public welfare departments actively promote it, with the government taking the lead." -13

IV. Key Factors associated with Donation Intentions

Qualitative analysis identified six major themes shaping participants' willingness to donate: (1) individual economic status, (2) altruism and social responsibility, (3)

Reciprocity after receiving benefits, (4) project perception and acceptance, and (5) trust in donation transparency (Table S8).

Table S8. Key Themes and Representative Quotes on Factors Influencing Donation Intentions

Themes	Code	Representative Quote
Individual economic status	● Ability to afford donations<br>● Within one's means<br>● Economic constraints limiting donation behavior	● *"My income is alright, so donating money doesn't really matter." - 09*<br>● *"And for us, being retired workers, I feel this small amount doesn't really matter. Our pensions aren't very low either." - 03*<br>● *"The first reason was that my spouse was ill, and due to our income, I donated ten yuan. Another reason was that I felt I could give a little within my own means." - 02*<br>● *"Of course, personal income affects the donation. How much to give really depends on each individual's situation." - 10*<br>● *"My family has financial difficulties." - 11*<br>● *"At the time, I did feel I should donate out of goodwill, but my financial circumstances didn't allow me to. My income isn't very high, and I have quite a heavy family burden. I really wanted to, and if I had the means, I would have donated." - 12*
Altruism and social responsibility	● Willingness to help others<br>● Sense of responsibility<br>● Spirit of mutual support<br>● Perceived contribution to society	● *"I take part in any kind of charitable activity." -05*<br>● *"Yes, I'm still willing to show my kindness." - 05*<br>● *"Because the help from society reduces or even prevents the possibility of getting pneumonia, we also have a responsibility to help — that is, according to our own ability or finances." - 01*<br>● *"Well, it's really about pulling together, isn't it? When someone's in trouble, people do step in to give a hand. The money might not be much, but once you put it all together, it can go a long way." - 04*
Reciprocity after receiving benefits	● Receiving subsidies increases willingness to donate<br>● Willingness to give back after receiving benefits<br>● Feeling personally benefited	● *"Because there was that 150 yuan support available then, I felt it was alright to make a small donation as well." -02*<br>● *"I wouldn't say it exactly like that. It's more that, once it was suggested, I thought it was right to give a little, just as a gesture." -07*<br>● *"Yes, it did make a difference. With the allowance in place, people, including myself, felt more willing to donate." -01*

		● *"Because I'd had this jab before, I knew how much it cost. So I thought, if I give a bit of money, to be honest, it still feels worthwhile. And since I had it, the effect was really good." -03*<br>● *"I think it's a good thing, and for me personally it's been beneficial. Putting in a bit of money to give some support is voluntary as well." -09*
Project perception and acceptance	● Positive perception of the project<br>● Acceptance of the project<br>● Opinions on donation approach	● *"My feeling is like this: the project itself is a good thing. For people like us older folks — I'm 76 this year — flu, pneumonia, and shingles are all things we're more likely to get. So when this project came along, and there was also a chance to make a small gesture myself, I welcomed it wholeheartedly." -01*<br>● *"I think it's a good thing, and it's still acceptable." -09*<br>● *"I feel this project should be initiated by the state, and it shouldn't require elderly patients to donate money. I don't think that's a very good approach." -06*
Trust in donation transparency	● Trust in donation management<br>● Lack of trust in the donation management	● *"I'm not worried about where my donation goes. I trust the organisers of this project (to manage and use our contributions properly). " -01*<br>● *"I' m not worried about the money. I trust the staff to use it just for what it' s meant for. " -02*<br>● *"As for where the donations go, I think the government is pretty strict about this nowadays, so usually there's no problem. I still trust quite a bit."-03*<br>● *"I've never really worried about it; not everyone is that bad....... Just putting the use of the money on the official WeChat account is enough, and I trust it a lot."-04*<br>● *"We never really thought about it. I trust the community quite a bit (to use our donations in right way)."-07*<br>● *"I don' t really trust where the donations come from or where they go." -06*
