# Supplementary material for: Pay-it-forward intervention increased pneumococcal vaccine uptake among older adults in China: a randomized controlled trial
Source: BMC Med. 2026 Jan 19;24:93. doi: 10.1186/s12916-026-04624-2 (PMC12895929; doi:10.1186/s12916-026-04624-2)
Supplement: Supplementary file 8 — Additional file 8. Odds ratios under hypothetical contamination scenarios. [file 12916_2026_4624_MOESM8_ESM.pdf]

Odds Ratios Under Hypothetical Contamination Scenarios

Contamination Proportion	Vaccinated in Control Group	Unvaccinated in Control Group	Odds Ratio (OR)
0	15	96	15.6
0.05	18.17	92.83	12.45
0.1	21.36	89.64	10.23
0.2	27.73	83.27	7.32
0.3	34.10	76.90	5.5
